# Pharmacokinetics of nikkomycin Z following multiple doses in healthy subjects

**DOI:** 10.1128/aac.00395-25

**Published:** 2025-06-06

**Authors:** David E. Nix, Susan E. Hoover, Nathan J. Hanan, John Galgiani

**Affiliations:** 1 Department of Pharmacy Practice & Science, R. Ken Coit College of Pharmacy, University of Arizona 8041, Tucson, Arizona, USA; 2 Department of Medicine, University of South Dakota, Sioux Falls, South Dakota, USA; 3 Clinical Pharmacology Modeling and Simulation, Glaxo Smith-Kline, Collegeville, Pennsylvania, USA; 4 Valley Fever Center for Excellence, Department of Medicine, Department of Immunobiology, College of Medicine-Tucson, and BIO5 Institute, University of Arizona 8041, Tucson, Arizona, USA; University Children's Hospital Münster, Münster, Germany

**Keywords:** nikkomycin Z, antifungal, pharmacokinetics, chitin synthase, *Coccidioides*

## Abstract

Nikkomycin Z is an investigational antifungal agent that inhibits chitin synthesis. The drug shows promise against endemic fungi such as *Coccidioides* spp. The purpose of this study was to determine the pharmacokinetics and safety when administered as multiple, ascending doses in healthy subjects. Healthy adult volunteers received nikkomycin Z in oral doses ranging from 250 mg twice daily to 750 mg three times a day for 14 days. An intensive pharmacokinetic study was conducted after the first dose (day 1) and after the last dose (day 14). Subjects were also monitored for safety and tolerance but were not confined to the facility continuously. Doses were tracked by self-reporting and were observed prior to intensive pharmacokinetic studies. On day 14, the mean (sd) maximal concentration ranged from 3.70 (1.08) to 6.89 (1.59) mg/L, and mean time of maximal concentration ranged from 2.3 to 3.0 h. The mean area under the time curve from time 0 to end of the dosing interval (8 or 12 h) was 17.3 (5.2) mg h/L for 250 mg twice daily, 28.5 (9.5) for 500 mg twice daily, 34.5 (10.9) for 750 mg twice daily, and 35.6 (8.4) for 750 mg thrice daily. The mean half-life ranged from 1.94 to 2.18 h. Bioavailability was less than proportional to dose for the 750 mg doses. Nikkomycin Z was well tolerated, and the study was completed without any serious safety concerns. This study supports continued development of nikkomycin Z as a potential therapeutic for the treatment of coccidioidomycosis.

## INTRODUCTION

Coccidioidomycosis is a fungal infection that occurs in limited desert areas of the Southwest United States, Northern Mexico, and Central and South America. The disease is caused by two closely related species, *Coccidioides immitis* and *C. posadasii*. Transmission is via inhalation of airborne arthroconidia from the environment. Once in the lung, the arthroconidia transition to a spherule (yeast form) and develop endospores. After about 4 days, the mature spherule ruptures and releases hundreds of endospores. The majority of infections are asymptomatic or associated with mild respiratory symptoms; however, severe and/or chronic lung disease may occur, and some patients develop disseminated infection to the meninges, bone, and visceral sites. A subset of patients exhibits chronic infection or infection-related complications that require long-term management, sometimes for life. Antifungal azoles are generally used for treatment, although amphotericin B may be used (1). Nikkomycin Z exhibits antifungal activity and is a promising agent being explored for the treatment of coccidioidomycosis.

Nikkomycin Z is a small-molecule antifungal agent consisting of a uridine-based nucleoside and a peptide component. This agent acts to inhibit chitin synthase (CHS), which is an enzyme integrated into the fungal cell membrane and is responsible for homopolymerization of N-acetyl glucosamine to form chitin (2). Chitin is a structural component of the cell wall along with beta (1,3) D-glucan, beta (1,6) D-glucan, and mannoproteins. Following the synthesis of chitin, the polymer is extruded across the cell membrane, which is also facilitated by CHS. Similar to penicillin binding proteins in bacteria, there are multiple types of chitin synthases that are active in different phases of growth and replication. Seven classes of CHS in fungi have been identified, and nikkomycin Z inhibits class I enzymes (3, 4).

The antifungal activity of nikkomycin Z is limited in spectrum, and the activity against *Coccidioides* spp*.* is most promising (5). Echinocandins inhibit the synthesis of β(1,3)-D-glucan, which leads to upregulation of chitin synthesis (6). Potential synergistic activity between echinocandins and nikkomycin Z, and azoles and nikkomycin Z requires further exploration (7, 8). Nikkomycin Z is effective in the intranasal mouse model of pulmonary coccidioidomycosis and has shown promise in the treatment of natural infection in dogs (9, 10). Nikkomycin Z was also demonstrated to be effective in the treatment of Coccidioides meningitis (11). A single-dose pharmacokinetic study in healthy subjects demonstrated oral bioavailability and excellent tolerance. Doses studied ranged from 250 to 2,000 mg orally in the fasting state. The area under the concentration-time curve from time 0 to time infinity [AUC(0–∞)] increased proportionally to a dose up to 500 mg. Compared to 250 mg, the relative bioavailability was 70% for 1,000 mg, and 42%–47% for 1,500–2,000 mg doses. The elimination half-life was 2.1–2.5 h and independent of dose (12). The current study was conducted to extend pharmacokinetic characterization to multiple dosing and gain more experience with safety and tolerability.

## MATERIALS AND METHODS

A multiple-rising dose pharmacokinetic study was conducted in healthy subjects. The study was approved by the University of Arizona IRB, and informed consent was obtained prior to participation. The subjects were screened within 14 days of day 1 by medical history, brief physical exam, 12-lead electrocardiogram, and clinical laboratory studies including complete blood count with differential, prothrombin time, activated partial thromboplastin time, serum chemistry profile, and urinalysis. Subjects had to be within 18–40 years of age of either sex. Females of childbearing potential were required to use hormonal contraceptives plus a barrier method or agree to be abstinent for the duration of the study. A serum pregnancy test was done at screening and end of study (day 16). A urine pregnancy test was performed on day 1 prior to dosing, and the result had to be negative.

Subjects arrived at the study center before 07:00 on day 1 after an overnight fast. A venous catheter was placed in a forearm vein for collection of blood samples. Screening laboratory tests were repeated prior to dosing (baseline), and a blood sample and urine sample were collected for analytical blanks. The first dose of medication was given between 8:00 and 9:00 with 6–8 ounces of water. There were four dose groups with eight subjects per group. The groups were enrolled sequentially and consisted of six subjects assigned to nikkomycin Z and two subjects assigned to placebo. At least four subjects were evaluated prior to dose escalation of each subsequent cohort. Doses included 250 mg every 12 h, 500 mg every 12 h, 750 mg every 12 h, and 750 mg every 8 h. All doses were provided as one, two, or three 250 mg capsule(s) or matching placebo capsule(s), as unit doses in foil packs. Treatment assignment (active versus placebo) was random using a randomization table provided to the investigational pharmacy. The pharmacy ensured that the appropriate treatment was provided to individual subjects and labeled with subject identity. Treatment (active versus placebo) was double-blind. Dosing was continued for 13 days with a final dose on the morning of day 14.

Intensive pharmacokinetic sampling was performed after the morning dose on days 1 and 14. Subjects were allowed to leave the study center after the last pharmacokinetic specimen was collected on day 1 and returned in the morning of days 2, 4, 7, and 10 for in-person check-in and on day 14 for the collection of steady-state pharmacokinetic specimens. Clinical laboratory testing was done on days 4, 7, 10, and 14. Unit doses of study medication were provided for self-dosing, and subjects were asked to complete a log of each dose taken and the datetime, in addition to recording any adverse events experienced. Subjects also had a telephone number to call if they had any concerning issues. On scheduled visits, the morning dose was observed, and the dose log was reviewed with each subject for doses since the last visit.

During intensive pharmacokinetic sampling on days 1 and 14, 11 blood samples (5 mL each) were then collected at 0 (predose), 0.25, 0.5, 0.75, 1.0, 1.5, 2.0, 3.0, 4.0, 6.0, and 8.0 h following the morning dose. If the patient was assigned to every 12 h dosing, an additional sample was collected at 12 h prior to the next dose. Predose pharmacokinetic sampling was performed during check-in visits prior to the morning dose on day 2, 4, 7, and 10. All blood samples for pharmacokinetics were collected using heparin-containing vials, and the vials were centrifuged at 3000 g for 10 min. The plasma was then separated and divided into two cryotubes and frozen. Subjects were asked to urinate prior to dose 1 and a portion of the urine was frozen to serve as an analytical blank. On day 14, subjects were instructed to urinate prior to the morning dose (not collected). Following the morning dose, all urine voids were collected for an entire dose interval (12 or 8 h). Each time the subject urinated, the entire void was added to a container that was maintained at 4–8°C. At the end of the collection interval, the volume of urine was measured, and two portions were added to cryotubes and frozen for storage. Plasma and urine samples were frozen at −20°C for less than 14 h and then transferred to −80°C until thawing for analytical processing. The end of the study visit was on day 16, at least 48 h after the last dose of nikkomycin Z. Subjects underwent an interim medical history, physical examination, 12-lead electrocardiogram, and final clinical laboratory testing.

Nikkomycin Z concentrations were measured in plasma and urine using high performance liquid chromotography (HPLC) with ultraviolet detection (263 nm). The plasma was thawed, then 50 µL of sodium dodecyl sulfate (SDS) was added to 250 µL of serum and vortex-mixed. The sample was ultrafiltrated using an ultrafiltration device (Centrifree, Merck, Darmstadt, Germany). Centrifugation was performed with 2,000 g force × 50 min at 4°C. The SDS displaced drug from protein binding, and after acidification with acetic acid, the sample was loaded onto the HPLC. The mobile phase consisted of heptane sulfonic acid and acetonitrile in a 775:225 part ratio.

Non-compartmental pharmacokinetic analysis was performed using Win Nonlin (Pharsight Inc, San Francisco, CA) and all statistical analysis was done with SAS (SAS Institute, Cary, NC). The maximum concentration (Cmax) and Tmax were determined from observed data. The area under the concentration-time curve (AUC) from time 0−t was determined using the trapezoidal method, where t is the time of last measurable concentration. The elimination rate constant (λ_z_) was the negative slope of ln(C) versus t after selecting the terminal linear portion of the curve. For day 1, the AUC(0–∞) was calculated as AUC(0−t)+C(t)/ λ_z_. For day 14, AUC from time 0 to tau [AUC(0−tau)] was determined as AUC(0−t) if t = tau or AUC(0−t) + $Ct\;\frac{\left(1-e^{-\lambda z\;\left(tau-t\right)}\right)}{\lambda z}$ if a concentration is not available at t = tau. Pharmacokinetic parameter values were summarized using descriptive statistics. For day 1 data, AUC(0−∞) normalized to a 250 mg dose was presented in a scatter plot and subjected to linear regression. For day 14, AUC(0−tau) normalized to a 250 mg dose was evaluated in the same way. Dose proportionality was also assessed using the power model (13). Relatively, bioavailability was assessed by comparing the AUC per unit of dose. Renal clearance was estimated as amount recovered in urine divided by AUC in plasma for the corresponding time interval. The percentage of dose recovered in urine in a dosing interval was calculated on day 14. All other data, such as trough concentration on days 2, 4, 7, and 10, were presented descriptively.

## RESULTS

Assay inter-day precision (%RSD) was 3.08%, 3.93%, and 3.37% at quality control (QC) concentrations of 0.35, 2.00, and 6.00 µg/mL, respectively. Similar specifications were used for the urine assay, except that sample (urine) was diluted 1:10 with 0.2 M acetic acid, centrifuged at 3,000 g × 10 min, and then loaded onto the HPLC. Inter-day precision for urine (%RSD) was 1.90%, 5.74%, and 1.42% at QC concentrations of 15, 125, and 375 µg/mL, respectively.

Considerable efforts were made to ensure that subjects took doses as per protocol, given that all doses were not observed. Only 1% (8 of 752) of doses were reported as missed. Doses were reported as given more than 60 min off schedule for 9.6% of the doses, and none of these events happened for the dose preceding the pharmacokinetic study on day 14. All doses associated with intensive pharmacokinetic studies were observed.

**Fig 1:**
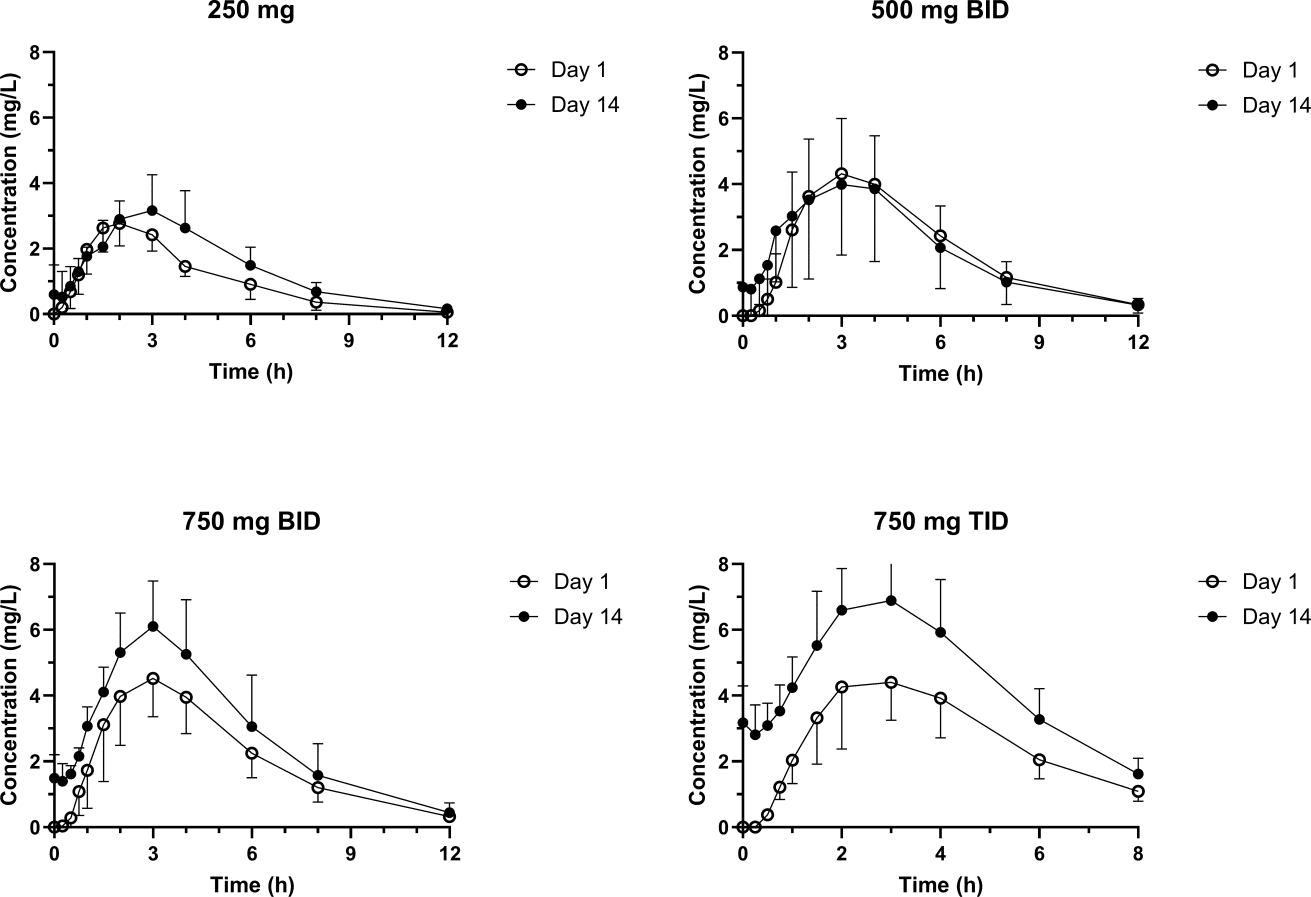
Nikkomycin Z plasma concentration-time profiles for day 1 and day 14. The figure is divided into four panels to cover the different dose levels including 250 mg every 12 h, 500 mg every 12 h, 750 mg every 12 h, and 750 mg every 8 h.

**Fig 2:**
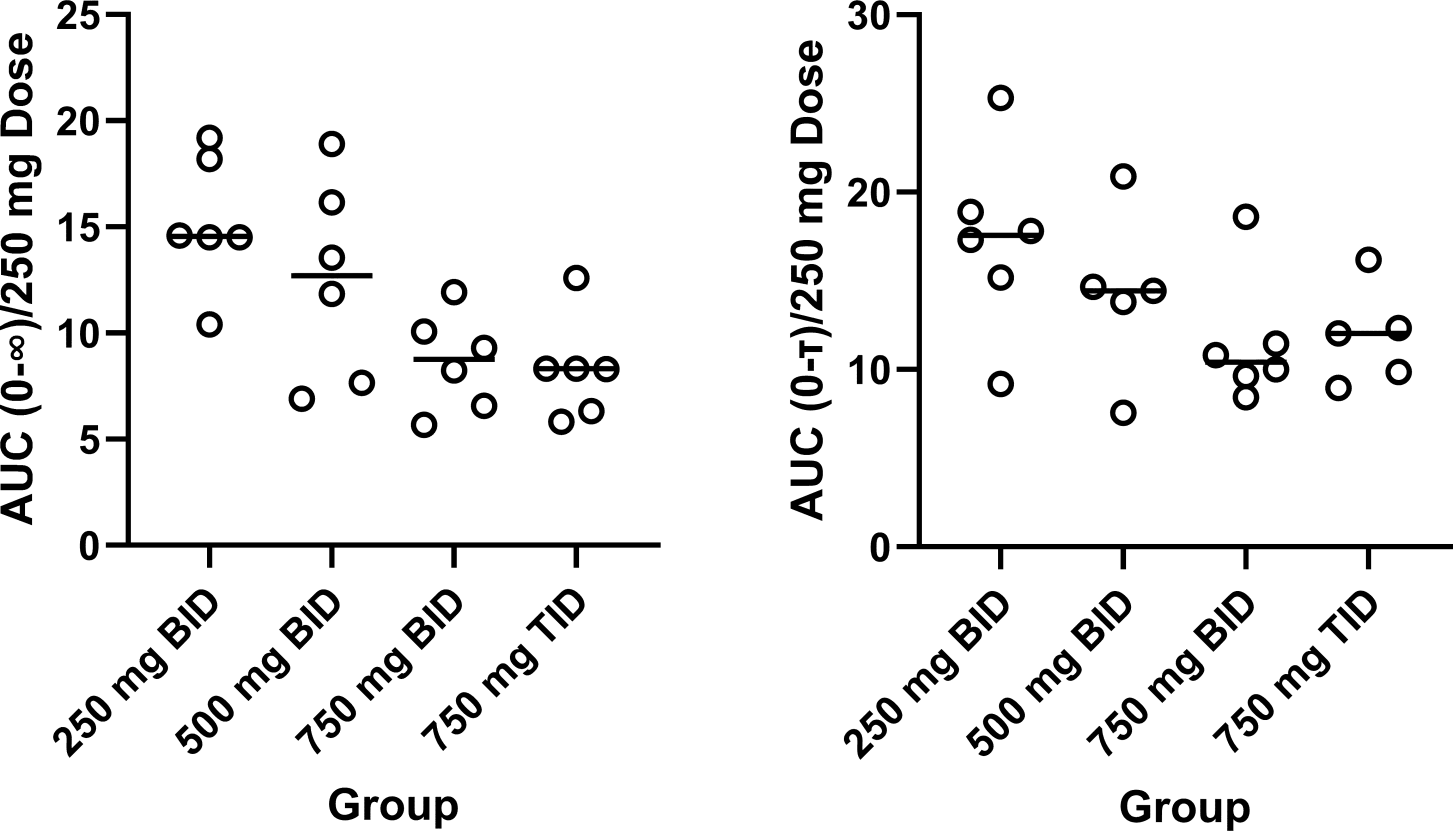
Dose normalized nikkomycin Z AUC for day 1 (left) and day 14 (right) for the four different dosing groups. All AUC values are normalized to a 250 mg dose, and the dose group refers to the study group. AUC(0–∞) and AUC(0–tau) are presented for day 1 and 14, respectively. The horizontal lines represent the mean values for each group.

Demographic characteristics of the study population are presented in Table 1. There were 17 women and 15 men enrolled. The mean (± sd) age overall was 32 (7.3) years, mean weight was 81.1 (21.3) kg, and mean BMI was 31 (27.9). The mean plasma concentrations versus time profiles are shown in Fig. 1. There was only slight accumulation with the 12-h dosing, but accumulation was notable with every 8 h dosing. Mean pharmacokinetic parameters are provided in Tables 2 and 3, respectively, for days 1 and 14. Figure 2 shows the relationship between AUC normalized to a 250 mg dose and dose level. Plasma exposure is dose-dependent and with no change in elimination rate; this suggests a decrease in bioavailability with increasing dose. There was a small accumulation noted on multiple dosing, and this was slightly greater than expected based on half-life. For dose proportionality using the power model (regression of ln(AUC) versus dose), the slope (90% CI) for day 1 was 0.447 (0.219–0.676) and for day 14 was 0.654 (0.422–0.885). The critical range for proportionality was 0.797–1.20. Similar findings were observed for Cmax, where the slope was 0.486 (0.207–0.765) for day 1 and 0.496 (0.257–0.735) for day 14. The conclusion is that nikkomycin Z bioavailability is proportional over a dose range of 250–500 mg but not proportional at higher doses.

**TABLE 1:** Demographic characteristics of study population^*a*^

Dose group	250 mg BID	500 mg BID	750 mg BID	750 mg TID	Placebo
Male/female	2/4	4/2	1/5	4/2	4/4
Race					
African American	0	0	1	0	0
Asian	0	0	0	0	1
White/Hispanic	3	2	2	2	1
White/non-Hispanic	3	4	3	4	6
Age (y)	27.8 ± 8.40	30.5 ± 8.96	30.7 ± 6.25	29.0 ± 7.51	31.8 ± 7.32
Weight (kg)	79.5 ± 14.1	94.1 ± 23.1	90.7 ± 31.6	74.4 ± 11.5	69.6 ± 15.2
BMI	28.1 ± 7.74	29.3 ± 9.09	35.6 ± 15.8	24.4 ± 1.51	22.9 ± 2.28

^*a*^ BID = every 12 h, TID = every 8 h. Values other than counts are presented as mean±sd.

**TABLE 2:** Nikkomycin Z pharmacokinetic parameters for day 1^*a*^

Group	Cmax (µg/mL)	Tmax (h)	Auc(0–∞) (µg h/mL)	T ½ (h)	CL/F (L/h)	V/F (L)
250 mg BID	2.81 ± 0.630	2.7 ± 0.52	15.2 ± 3.1	2.06 ± 0.148	17.9 ± 3.87	51.4 ± 15.8
500 mg BID	4.55 ± 1.63	3.2 ± 0.78	25.0 ± 9.4	2.10 ± 0.265	22.9 ± 9.48	68.6 ± 22.9
750 mg BID	4.72 ± 1.23	3.0 ± 0.63	25.9 ± 6.9	2.18 ± 0.221	30.9 ± 8.69	97.4 ± 31.1
750 mg TID	4.78 ± 1.61	2.6 ± 0.50	24.9 ± 7.1	2.17 ± 0.147	32.0 ± 8.14	101 ± 27.9

^*a*^ Cmax = maximum concentration, Tmax = time of maximum concentration, AUC (0–∞) = area under the plasma concentration-time curve from time 0 to time infinity, T ½ = terminal half-life, CL/F = oral clearance, and V/F = volume of distribution divided by fraction absorbed. All values are mean ± sd. BID = every 12 h, and TID = every 8 h.

**TABLE 3:** Nikkomycin Z pharmacokinetic parameters for day 14^*a*^

Group	Cmax (µg/mL)	Tmax (h)	AUC(0–tau) (µg h/mL)	T ½ (h)	CL/F (L/h)	V/F (L)
250 mg BID	3.70 ± 1.08	2.3 ± 0.61	17.3 ± 5.2	1.94 ± 0.228	15.8 ± 5.90	43.5 ± 12.5
500 mg BID	5.02 ± 1.29	2.7 ± 0.49	28.5 ± 9.5	2.15 ± 0.215	19.5 ± 7.95	59.2 ± 19.9
750 mg BID	6.23 ± 1.24	2.7 ± 0.51	34.5 ± 10.9	2.11 ± 0.176	23.2 ± 5.46	69.5 ± 12.7
750 mg TID	6.89 ± 1.59	3.0 ± 0.03	35.6 ± 8.40	2.18 ± 0.465	21.9 ± 4.84	69.8 ± 24.6

^*a*^ Cmax = maximum concentration, Tmax = time of maximum concentration, AUC (0–tau) = area under the plasma concentration-time curve from time 0 to end of dosing interval (8 or 12 h), T ½ = terminal half-life, CL/F = oral clearance, and V/F = volume of distribution divided by fraction absorbed. All values are mean ± sd. BID = every 12 h and TID = every 8 h.

Nikkomycin Z percent of dose excreted in urine for the dosing interval on day 14 averaged 45.7% (16.4) for 250 mg every 12 h, 32.1% (8.3, *n* = 5) for 500 mg every 12 h, 28.6% (6.3) for 750 mg every 12 h, and 33.3% (16.6, *n* = 4) for 750 mg every 8 h. Mean renal clearance values for the same groups were 112 (22.3), 96.1 (15.5), 111 (33.1), and 118 (53.7) mL/min, respectively.

There were 57 adverse events in 24 subjects who received nikkomycin Z and 14 events in 8 subjects who received placebo. Events occurring in three or more subjects included headache, diarrhea, and pre-syncope. Headache occurred in 11 of 24 (45.8%) subjects on active treatment and 5 of 8 (62.5%) on placebo. Diarrhea was noted in 2 of 24 subjects on active treatment and 1 of 8 subjects on placebo. There were three cases of pre-syncope which occurred only in subjects receiving nikkomycin Z; however, the events were judged as related to study procedures such as blood collection and fasting. There were no serious adverse events, non-sporadic laboratory changes, or ECG changes that were temporally related to nikkomycin exposure.

## DISCUSSION

The only comparable pharmacokinetic data in humans are from the single, rising dose study (9). The mean AUC(0–∞) following a single dose normalized to 250 mg was 11.6–15.2 (250 mg), 12.3–12.5 (500 mg), 8.3–8.6 (750 mg), 8.1 (1,000 mg), 5.1 (1,500 mg), 5.4 (1,750 mg), and 5.1 µg h/mL (2,000 mg). Drug exposure is not proportional to dose administered, and there is a drop in bioavailability of about 35% with doses greater than 500 mg (750 or 1,000 mg) and 60% with doses greater than 1,000 mg (1,500–2,000 mg). Nikkomycin Z is a hydrophilic compound and probably fits into Biopharmaceutics Classification System Class III, including drugs with high solubility and low permeability. There is an opportunity for optimization of drug delivery, including administration with food to slow gastric emptying and optimization of drug formulation (14).

With the highest dose in this study (750 mg every 8 h), 24 h AUC averaged 107 µg h/mL (35.6 from Table 3 x 3), and no dose-limiting toxicity was observed. In mice, the model predicted dosing of nikkomycin Z at 44 mg/kg/day would result in 90% of the maximum observed reduction in fungal burden. Administration of a 40 mg/kg single subcutaneous dose of nikkomycin Z in mice provided an average AUC(0–∞) of 27.2 µg h/mL (9). Thus, exposure to approximately 30 µg h/mL is required for a 90% reduction in fungal burden in the mouse intranasal infection model. Nikkomycin Z administration to several naturally infected dogs resulted in improvement or resolution of disease. The typical nikkomycin Z exposure in this cohort of dogs was 24 h AUC of 38.3 µg h/mL (10). Considering these experimental results, nikkomycin Z is a promising drug to treat coccidioidomycosis. This study contributes that exposures following oral dosing that are associated with positive responses in mice and dogs can be achieved in humans without observed dose-limiting toxicity or serious adverse events.

### Conclusion

Nikkomycin Z with doses of 250 mg every 12 h to 750 mg every 8 h achieves exposure in humans that is within the range of 24 h AUC associated with efficacy in mice and dogs. Exposure is less than proportionally increased relative to dose with doses greater than 500 mg. No dose-limiting or serious adverse events were observed.
